# Transient Pulses and Transcriptomic Rigidity: Dynamic miRNA Expression Patterns in Ovine Ovarian Development Reveal Molecular Strategies Underlying Seasonal Reproduction

**DOI:** 10.3390/biology15201828

**Published:** 2026-10-13

**Authors:** Ruchao Tian, Yingyue Lv, Yuqian Chen, Zhidan Wang, Anqi Wang, Bo Gu, Huaizhi Jiang

**Affiliations:** 1 College of Life Science, Jilin Normal University, Siping 136000, China; 2 College of Animal Science and Technology, Jilin Agricultural University, Changchun 130118, China

**Keywords:** Ujumqin sheep, ovarian development, miRNA, ceRNA, transient pulse pattern

## Abstract

Ovine fecundity is a key economic trait constraining livestock production efficiency, and the marked differences in reproductive strategies among breeds provide an ideal model for dissecting the molecular mechanisms underlying seasonal reproduction and prolificacy. In this study, we investigated Ujumqin sheep, a typical seasonally breeding, single-litter breed, by collecting ovarian tissues at three critical developmental stages—3 months (pre-pubertal), 6 months (puberty initiation), and 12 months (sexual maturity)—and performed whole-transcriptome sequencing to systematically characterize the dynamic expression trajectories of miRNAs, lncRNAs, circRNAs and mRNA during ovarian development. Unexpectedly, we found that miRNA expression changes did not follow a monotonic linear trajectory, but rather exhibited a highly nonlinear “transient pulse” pattern: pairwise comparisons revealed 42 and 44 differentially expressed miRNAs (DEMs) between 3 vs. 6 months and 6 vs. 12 months, respectively, yet only 21 DEMs between 3 vs. 12 months—an “inverted-V” distribution. Cross-validation by Mfuzz temporal clustering and Venn diagram intersection analyses pinpointed 10 core miRNAs that synchronously elevated during the puberty initiation window (3 → 6 months) and returned to baseline by sexual maturity (6 → 12 months). Target gene enrichment of these 10 miRNAs converged significantly in Wnt, cAMP, FoxO, and oxytocin signaling pathways—suggesting that these miRNAs are associated with molecular programs related to follicular development and hormone signaling at the 6-month puberty initiation window. We further constructed a competing endogenous RNA (ceRNA) regulatory network centered on these 10 core miRNAs and resolved two potential functional axes, lncRNA MSTRG.20588-miR-211-CTNNB1 and circRNA3387-miR-320d-ATG7, directly linking the transient pulse signals to follicle dominance selection and granulosa cell quality control. An exploratory Cross-breed comparison revealed that the prolific breed Small-Tailed Han sheep exhibited 227 DEMs at the puberty initiation window (approximately 5.4-fold more than Ujumqin sheep), with the early changes largely sustained into adulthood. In contrast, the majority of differential changes observed in Ujumqin sheep at 6 months were reversed and essentially “reset” by 12 months. Based on these findings, we propose a conceptual framework of “transcriptomic rigidity” versus “transcriptomic plasticity” to distinguish the two breeds: Ujumqin sheep ovaries tend to reset the transcriptome to a quiescent state after completing puberty initiation—a “rigidity” strategy that may represent the molecular hallmark of seasonal breeding breeds. Collectively, this study provides new conceptual tools for understanding the molecular regulation of seasonal reproduction in sheep and offers potential molecular targets for genetic improvement of reproductive performance in single-litter breeds.

## Simple Summary

Some sheep breeds give birth only once a year in a narrow season, while others can breed year-round and produce multiple litters. Understanding the molecular basis of this difference is important for improving livestock production. We studied Ujumqin sheep, a seasonal single-litter breed, by collecting ovaries at three developmental stages—before puberty, at puberty onset, and at sexual maturity—and sequenced all RNA molecules. Unexpectedly, small regulatory RNAs did not change gradually but rose together at puberty and then fell back by maturity, forming a brief pulse. Their target genes control follicle development and hormone signaling, indicating they are associated with molecular programs at the 6-month puberty initiation window. We identified two regulatory axes linking this pulse to follicle selection and egg quality. Compared with the prolific Small-Tailed Han sheep, which maintained many gene changes into adulthood, Ujumqin sheep reversed most changes and returned to a quiet state. These findings suggest that the two breeds differ in the timing and magnitude of ovarian gene expression changes during development, which may relate to their distinct reproductive strategies; moreover, they reveal how seasonal breeding is controlled at the molecular level and provide targets for improving reproduction in single-litter sheep.

## 1. Introduction

Fecundity is a core economic trait in sheep production that strongly influences production efficiency and directly affects farm profitability and breed competitiveness [1]. China possesses a rich diversity of indigenous sheep breeds; however, marked differences exist in their reproductive strategies. Ujumqin sheep, a locally adapted meat breed known for its hardiness and tolerance to coarse feed, has a seasonal reproductive pattern, with breeding restricted to autumn and typically one lamb per year, which constrains overall reproductive efficiency [2]. Unraveling the molecular underpinnings of this reproductive constraint is not only a theoretical imperative for advancing molecular breeding programs in sheep but also a practical challenge of immediate relevance—one that holds the key to breaking the seasonal breeding bottleneck and improving reproductive output in single-litter breeds.

The ovary is a key effector organ within the hypothalamic–pituitary–gonadal (HPG) axis in sheep, and its developmental trajectory determines follicular reserve, steroidogenic capacity, and ovulatory competence, while its activity is regulated by hypothalamic and pituitary signals. From puberty initiation to sexual maturation, the ovary undergoes a series of intricate morphological and functional remodeling events [3]. This process is precisely orchestrated by the HPG axis [4], and accumulating evidence in recent years has consistently unveiled that non-coding RNAs (ncRNAs), particularly miRNAs, play a regulatory role that far surpasses earlier expectations. Acting at the post-transcriptional level, miRNAs modulate target gene expression and are extensively implicated in key reproductive processes including ovarian granulosa cell proliferation [5,6], folliculogenesis and atresia [7], and steroid hormone biosynthesis [8,9]. In studies investigating puberty initiation in goats, high-throughput sequencing has successfully identified a large repertoire of DEMs [10]. Similarly, comparative analyses across distinct ovine ovarian developmental stages have pinpointed the oar-let-7 family (e.g., let-7a, let-7b) and oar-miR-148a as critical regulators of ovarian development [11]. Furthermore, lncRNAs and circRNAs function as molecular “sponges” for miRNAs, participating in multilayered regulation of ovarian development through the ceRNA mechanism, and multiple ceRNA networks associated with reproductive performance have been successfully constructed. For instance, the lncRNA SLC19A1-AS has been demonstrated to act as a ceRNA by sponging miR-1343, thereby relieving miR-1343-mediated repression of WNT11 and positively regulating granulosa cell proliferation in goats—a finding that established the SLC19A1-AS/miR-1343/WNT11 axis as a novel theoretical foundation for breeding high-fertility goats [12]. In another multi-omics study, extensive differentially expressed transcripts were identified in the ovaries of Chuanzhong black goats (prolific) versus Tibetan goats (monoestrous), and five critical ceRNA pairs were constructed, providing fresh insights into the molecular basis of goat prolificacy [13]. Additionally, a genome-wide circRNA sequencing study in Small-Tailed Han sheep and Duolang sheep identified 44 DECs and predicted several key ceRNA regulatory pairs linked to reproduction, such as circRNA_3257-novel579_mature-EPHA3-19-32 [14]. Although ovarian miRNAs, lncRNAs, circRNAs, and mRNAs have been studied in sheep and goats, most available datasets profile only a single RNA class or a limited number of stages. Consequently, the coordinated changes among miRNAs, mRNAs, lncRNAs, and circRNAs across the full trajectory from prepuberty to sexual maturity remain unclear. Most comparative studies have used cross-sectional comparisons of breeds or stages, which can identify differences but cannot reveal whether these differences form continuous developmental trajectories. In addition, the degree to which ovarian ceRNA networks are conserved across sheep breeds, and which modules are associated with seasonal, single-bearing reproduction, remain unclear.

Ujumqin sheep are an indigenous Mongolian-lineage meat breed from the Ujumqin steppe of Inner Mongolia, China. Ewes are seasonal breeders: under natural photoperiod, oestrous activity is restricted to the autumn breeding season when day length shortens, and most ewes produce one lamb per year [15]. This reproductive trait necessitates that, following the completion of puberty initiation, the ovary maintains a functional quiescent state during unfavorable seasons rather than sustaining continuous activation. This prompts a central question: what dynamic pattern do miRNAs assume during the transition from puberty initiation to sexual maturation in the ovary of Ujumqin sheep, and how does this pattern logically relate to their seasonal, monoestrous breeding strategy? To address this question, we selected three pivotal developmental time points—3 months (pre-pubertal), 6 months (puberty initiation), and 12 months (sexual maturity)—and performed whole-transcriptome sequencing to systematically delineate the dynamic expression landscapes of miRNAs, lncRNAs, circRNAs and mRNAs throughout ovarian development. Building upon this, we integrated target gene functional enrichment and ceRNA network analyses to dissect the regulatory networks governed by core miRNAs. Finally, through a comparative analysis with the corresponding developmental-stage miRNA dynamics of Small-Tailed Han sheep (a prolific breed), we further interrogated whether the molecular strategy manifested by Ujumqin sheep is breed-specific. Because all animals were kept under natural photoperiod, age and photoperiodic history are not independent; we therefore interpret the results as stage-associated ovarian transcriptome dynamics in a seasonal breed, not as photoperiod-specific effects. Taken together, the present findings are expected to provide a developmental baseline and data resource for ovarian maturation in seasonal sheep breeds and to identify candidate breed-specific regulatory nodes for future functional validation.

## 2. Materials and Methods

### 2.1. Sample Collection

The Ujumqin sheep used in this study were obtained from Guofeng Livestock Breeding Farm (Songyuan City, China). All experimental animals were healthy female sheep with no history of disease, raised under uniform management conditions with natural grazing, ad libitum access to feed and water, and exposure to natural photoperiod. Based on the growth and developmental characteristics of the breed, healthy ewes (n = 3 per time point) were selected at three critical stages: 3 months of age (pre-pubertal) (Utm1, Utm2, Utm3), 6 months of age (puberty initiation) (Usm1, Usm2, Usm3), and 12 months of age (sexual maturity) (Ud1, Ud2, Ud3). The age variation among individuals at each time point was controlled within ±5 days, and body weights fell within the normal range for the breed and age, thereby minimizing the potential confounding effects of individual variation and environmental factors on subsequent analyses. Because sampling was performed on a single date, season and photoperiod were identical across the three groups. Birth dates differed among age groups; therefore, postnatal photoperiodic history inevitably differed with age, and this limitation is explicitly acknowledged in the Discussion. Animals within each timepoint were unrelated: each was born to a different dam and sired by a different ram, with no common ancestry within three generations according to breeding records. On the morning of sampling, animals were euthanized under fasting conditions, after which the abdominal cavity was immediately opened to expose the reproductive tract. Both ovaries were excised intact, promptly snap-frozen in liquid nitrogen, and transported to the laboratory for storage until further processing. All experimental procedures were approved by the Animal Ethics Committee of Jilin Normal University (KJLL20260809).

### 2.2. RNA Extraction, Library Construction, and Illumina Sequencing

Approximately 100 mg of ovarian tissue from Ujumqin sheep stored at −80 °C was ground into a fine powder in a liquid nitrogen-precooled mortar. Total RNA was extracted using TRIzol^®^ reagent (Invitrogen, Carlsbad, CA, USA). RNA concentration and purity were assessed with a NanoDropTM 2000 spectrophotometer (Thermo Fisher Scientific, Wilmington, DE, USA), and samples with an A260/A280 ratio between 1.8 and 2.2 were considered acceptable. RNA integrity was evaluated using an Agilent 2100 Bioanalyzer system (Agilent Technologies, Santa Clara, CA, USA), and only samples with an RNA Integrity Number (RIN) ≥ 7.0 were used for subsequent library construction.

Whole-transcriptome sequencing libraries were prepared in two separate workflows. For large RNA species, including mRNA, lncRNA, and circRNA, a strand-specific library preparation strategy with ribosomal RNA (rRNA) depletion was employed. Small RNA libraries (covering miRNAs) were constructed using the TruSeq Small RNA Sample Prep Kit (Illumina, San Diego, CA, USA). PCR amplification was performed for 12–15 cycles, and size selection was carried out by PAGE gel purification to enrich fragments of 18–26 nt. Following quality control, the strand-specific libraries were sequenced on an Illumina NovaSeq 6000 platform (Illumina Inc., San Diego, CA, USA) in paired-end mode (PE150) with a read length of 150 bp. The small RNA libraries were sequenced on an Illumina HiSeq 2500 platform (Illumina Inc., San Diego, CA, USA)in single-end mode (SE50) with a read length of 50 bp.

### 2.3. Data Quality Control and Filtering

For large RNA sequencing data, the quality control procedure was performed as follows: (1) cutadapt (v1.10) was used to remove adapter sequences and primer contamination; (2) bases with a quality score below 3 at the 5′ end and below 15 at the 3′ end were trimmed, and reads with an average quality score below 15 were discarded using a sliding window approach (window size: 4 bp); (3) reads shorter than 36 bp in length were filtered out; (4) reads containing more than 10% ambiguous bases (Ns) were removed.

For small RNA sequencing data, the quality control procedure was as follows: (1) cutadapt (v1.10) [16] was used to trim the 3′ adapter sequences; (2) reads shorter than 18 nt or longer than 26 nt were discarded; (3) reads containing more than 10% ambiguous bases (Ns) were removed; (4) low-quality reads were excluded (those in which the proportion of bases with a quality score Q ≤ 5 exceeded 50% of the read length). The high-quality clean reads obtained after quality control were used for subsequent bioinformatics analyses.

### 2.4. Read Alignment and Transcript Identification

For large RNA sequencing data (with mRNA, lncRNA, and circRNA sharing the same alignment results), read alignment was performed using HISAT2 (v2.2.1) [17,18,19]. Transcript assembly was carried out with StringTie (v2.1.6) [19,20,21], using the BAM alignment files from each sample as input and the reference genome annotation file (GTF) as a guide, and assemblies were generated for each sample individually. Following transcript assembly, gffcompare was used to compare the assembled transcripts against the reference genome annotation to identify known mRNAs. For previously unannotated transcripts, coding potential was further assessed using two complementary programs, CPC (v0.9-r2) and CNCI (v2.0). Transcripts with coding potential were classified as novel mRNAs; those predicted to lack coding potential were further filtered by discarding transcripts shorter than 200 nt, and the remaining transcripts were designated as candidate lncRNAs.

CircRNA identification was performed based on reads spanning backspliced junctions (BSJ) within the large RNA sequencing data. The quality-filtered clean reads were first aligned to the sheep reference genome using TopHat2 (v2.1.0), and reads that did not map to the reference genome were subjected to tophat-fusion for non-linear alignment. CircRNAs were subsequently identified using the circRNA detection software CIRCexplorer2 [22]. Concurrently, CIRI2 (v2.0.6) [23] was employed as an independent circRNA identification tool. The final circRNA set was defined as the intersection of CIRCexplorer2 and CIRI2 predictions, and only circRNAs supported by at least 2 independent BSJ-spanning reads with at least 8 bp anchored on each side of the junction were retained.

For small RNA sequencing data, miRNA identification was performed using the ACGT101-miR (v4.2) pipeline. Briefly, the quality-filtered small RNA clean reads were sequentially aligned to the Rfam database (v12.0, covering ncRNAs such as rRNA, tRNA, snRNA, and snoRNA) and the Repbase repetitive sequence database (v22.07) to remove non-miRNA reads. The remaining reads were then aligned to the sheep reference genome (ARS-UI_Ramb_v3.0) and known miRNA precursor sequences. Known miRNAs were identified based on mature miRNA sequences from sheep and closely related species in the miRBase database (v22.1), allowing no more than two mismatches. Sequences that could not be annotated as known miRNAs were subjected to secondary hairpin structure prediction to identify potential novel miRNAs. miRNA expression levels were quantified as transcripts per million (TPM) normalized reads.

### 2.5. Differential Expression and Temporal Dynamic Analyses

Differential expression analysis was performed using two complementary strategies. (1) For pairwise differential expression analysis, DESeq2 (v1.22.2) [24,25] was applied to perform pairwise comparisons among the three time points, namely Utm vs. Usm (Set 1), Usm vs. Ud (Set 2), and Utm vs. Ud (Set 3). The thresholds for significant differential expression were set at |log_2_ (fold change)| ≥ 1 and padj < 0.05. Venn diagrams were generated to analyze the overlaps among the DEMs from the three comparison sets, aiming to identify miRNAs that commonly responded across the developmental transitions. (2) For global differential testing across multiple groups, ANOVA was employed to identify miRNAs exhibiting overall expression fluctuations throughout the entire developmental course, with FDR < 0.05 as the significance threshold to screen for miRNAs with significant overall expression differences among the three time points.

For temporal dynamic analysis, fuzzy C-means clustering was performed using the Mfuzz package (v2.54.0). Briefly, the procedure comprised the following steps: (1) miRNAs with missing values in >25% of samples were removed, and the remaining missing values were imputed with the mean; (2) low-variation miRNAs with standard deviation < 0.5 were excluded; (3) expression values of each miRNA across the three time points were Z-score normalized to a mean of 0 and a standard deviation of 1 (Z-scores are used for visualization only, and that the actual TPM values and fold changes are available in Supplementary Table S5; (4) the fuzzification parameter m was estimated using the m-estimate function; (5) to balance clustering resolution and biological interpretability, parallel analyses were performed with cluster numbers set to c = 4 and c = 6 to evaluate clustering robustness and identify fine-grained expression subtypes; and (6) clustering was executed using the mfuzz function, and core miRNAs within each cluster were extracted using the acore function with a membership threshold ≥ 0.7.

### 2.6. Target Gene Prediction and Functional Enrichment Analysis

Target genes of miRNAs were predicted using miRanda (v3.3a) [26,27,28] and TargetScan (v5.0) [29,30,31], and the intersection of the two prediction sets (with thresholds set as TargetScan_score ≥ 50 and miranda_Energy < −10) was retained as the high-confidence target gene set. Functional annotation and pathway enrichment analyses of the target genes were performed using the Gene Ontology (GO) database (http://geneontology.org/) and the Kyoto Encyclopedia of Genes and Genomes (KEGG) database (http://www.genome.jp/kegg/ (accessed on 16 July 2026)), respectively. Significance was evaluated using Fisher’s exact test, with a threshold of *p* < 0.05 considered statistically significant.

### 2.7. ceRNA Network Construction

To elucidate the upstream regulatory mechanisms of the core miRNAs, we predicted the targeting relationships between the core miRNAs and the differentially expressed lncRNAs (DELs) and circRNAs (DECs) based on the ceRNA hypothesis. Parallel predictions were performed using TargetScan (v5.0) and miRanda (v3.3a), and only lncRNA/circRNA–miRNA interaction pairs identified by both software packages were retained. These interactions were then integrated to construct a ceRNA regulatory network. Finally, all ceRNA triplets were imported into Cytoscape (v3.10.1) for visualization.

### 2.8. Cross-Breed Comparative Analysis

To assess whether the dynamic patterns of ovarian miRNAs in Ujumqin sheep are breed-specific, we compared the number and dynamic pattern characteristics of DEMs detected at each developmental stage in Ujumqin sheep with ovarian sRNA sequencing data from Small-tailed Han sheep at 1, 6, and 12 months of age, which were previously obtained by our group using the same sequencing platform. Small-tailed Han sheep have high fecundity and multiple lambs per litter, whereas Ujumqin sheep have lower fecundity and usually produce a single lamb. Although both belong to the same species, their pronounced differences in reproductive phenotype make them a suitable comparison for studying ovarian developmental dynamics. DEMs were identified using consistent criteria in both breeds (|log_2_FC| ≥ 1, padj < 0.05), and the analysis focused on the number of DEMs and overlap size across corresponding developmental windows. This comparison helps distinguish shared programs of ovarian development from breed-specific programs potentially associated with fecundity differences, thereby providing a more accurate characterization of miRNA dynamics during ovarian development in Ujumqin sheep.

### 2.9. RT-qPCR Validation

To validate the reliability of the whole-transcriptome sequencing data, three DEMs and three differentially expressed mRNAs were randomly selected for expression level verification using real-time quantitative PCR (RT-qPCR). The primer sequences used are provided in Supplementary Table S1. Relative expression levels of the target molecules were calculated using the 2^−ΔΔCt^ method, and statistical analysis was performed using one-way ANOVA with SPSS software (v18.0), with significance thresholds as described above.

## 3. Results

### 3.1. Sequencing Data Quality Assessment

A total of nine ovarian tissue samples from Ujumqin sheep at 3 months of age (Utm1, Utm2, Utm3), 6 months of age (Usm1, Usm2, Usm3), and 12 months of age (Ud1, Ud2, Ud3) were subjected to RNA extraction and sequencing. The quality assessment of total RNA for all samples is presented in Supplementary Table S2, confirming that RNA concentrations and integrity met the requirements for subsequent experiments. For large RNA library sequencing (covering mRNA, lncRNA, and circRNA), a total of 741,763,382 raw reads were generated, with per-sample yields ranging from 71.5 to 92.5 million reads. After quality control, 717,581,340 valid reads were retained, with an average Q30 score of 97.89%. The average alignment rate to the sheep reference genome (ARS-UI_Ramb_v3.0) was 92.96% (ranging from 91.02% to 94.66%), as detailed in Supplementary Table S3. For small RNA library sequencing (covering miRNAs), a total of 99,719,749 raw reads were generated, with per-sample yields ranging from 9.9 to 11.4 million reads. After quality control, 83,411,504 clean reads were retained, with an average Q30 score of 97.13%. The alignment rates to the reference genome ranged from 88.39% to 99.32%, as shown in Supplementary Table S4.

Characterization of different RNA species revealed the following features: the insert sizes of the mRNA/lncRNA libraries were predominantly concentrated in the 200–300 bp range, with uniform gene coverage and the proportion of reads mapping to exonic regions conforming to expectations (Supplementary Figure S1). The small RNA libraries displayed read length distributions strictly centered at 21–23 nt, and the 5′ terminal nucleotide bias (uracil preference) was consistent with the canonical biogenesis features of small RNAs (Supplementary Figure S2). For circRNA detection, the identified circRNAs were predominantly longer than 1000 nt, which is consistent with the typical length distribution of circRNAs. In terms of genomic origin, exonic circRNAs constituted the largest proportion, in agreement with the canonical model of circRNA formation via back-splicing (Supplementary Figure S3).

Collectively, these results demonstrate that all types of RNA libraries were successfully constructed and that the sequencing data were of high quality, proving adequate for subsequent differential expression and functional enrichment analyses.

### 3.2. Differential Expression Dynamics During Ovarian Development

**Figure 1:**
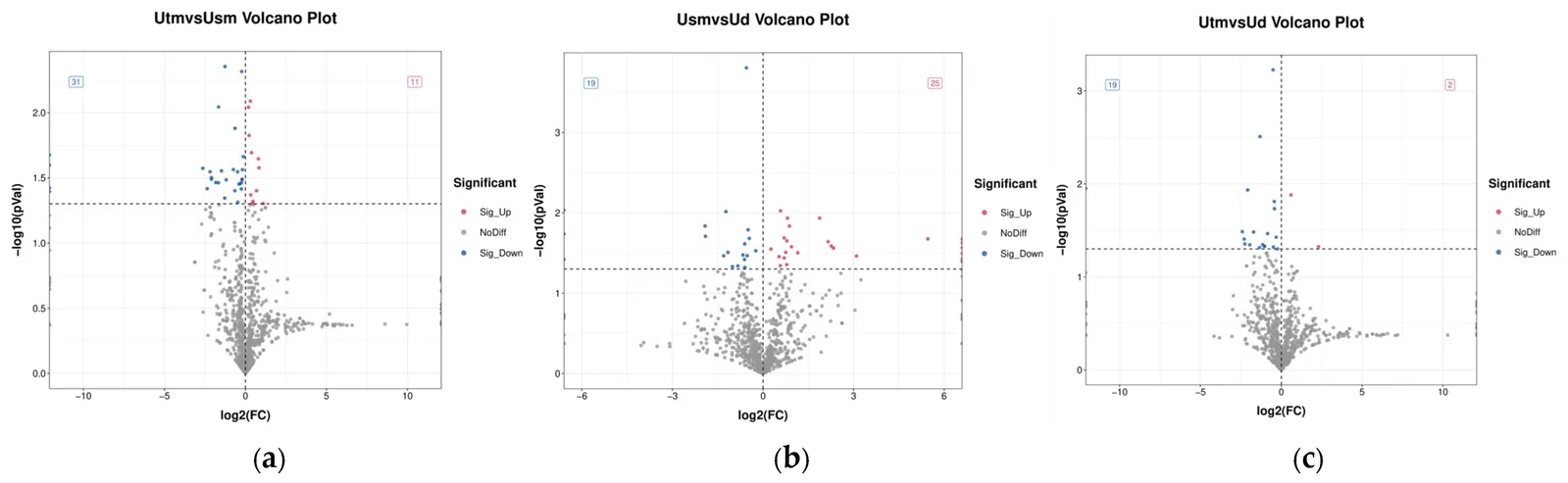
Volcano plot of DEMs: (**a**) Utm vs. Usm; (**b**) Usm vs. Ud; (**c**):(**a**) Utm vs. Ud.

To delineate the dynamic transcriptomic changes accompanying ovarian development in Ujumqin sheep, we performed pairwise differential expression analyses across the three developmental stages: Utm, Usm and Ud. As summarized in Figure 1, the number of dDEMs identified in each comparison was as follows: Utm vs. Usm (Set 1) yielded 42 DEMs (11 up-regulated and 31 down-regulated); Usm vs. Ud (Set 2) yielded 44 DEMs (25 up-regulated and 19 down-regulated); and Utm vs. Ud (Set 3) yielded 21 DEMs (2 up-regulated and 19 down-regulated). The corresponding numbers for differentially expressed circRNAs (DECs) were 178 (104 up, 74 down) in Set 1, 213 (170 up, 43 down) in Set 2, and 170 (133 up, 37 down) in Set 3 (Supplementary Figure S4). For lncRNAs, the counts were 386 (174 up, 212 down) in Set 1, 364 (222 up, 142 down) in Set 2, and 432 (258 up, 174 down) in Set 3 (Supplementary Figure S5). For mRNAs, the numbers were 3616 (1975 up, 1641 down) in Set 1, 3629 (1852 up, 1777 down) in Set 2, and 3538 (1989 up, 1549 down) in Set 3 (Supplementary Figure S6). Unexpectedly, the DEM counts exhibited a distinctly non-linear distribution pattern: 42 DEMs between Utm and Usm, 44 between Usm and Ud, but only 21 between Utm and Ud. From a developmental biology perspective, one would anticipate the greatest phenotypic divergence between the pre-pubertal (3 months) and adult (12 months) stages, and consequently the largest number of molecular differences. However, the Utm vs. Ud comparison yielded far fewer DEMs than either transitional comparison—and substantially fewer than conventional expectations would predict. This “inverted-V” shaped distribution suggests that the ovary of Ujumqin sheep undergoes a pronounced miRNA “pulsatile perturbation” during the puberty initiation window at 6 months, with the majority of these perturbed signals being systematically withdrawn by the adult stage at 12 months.

**Figure 2:**
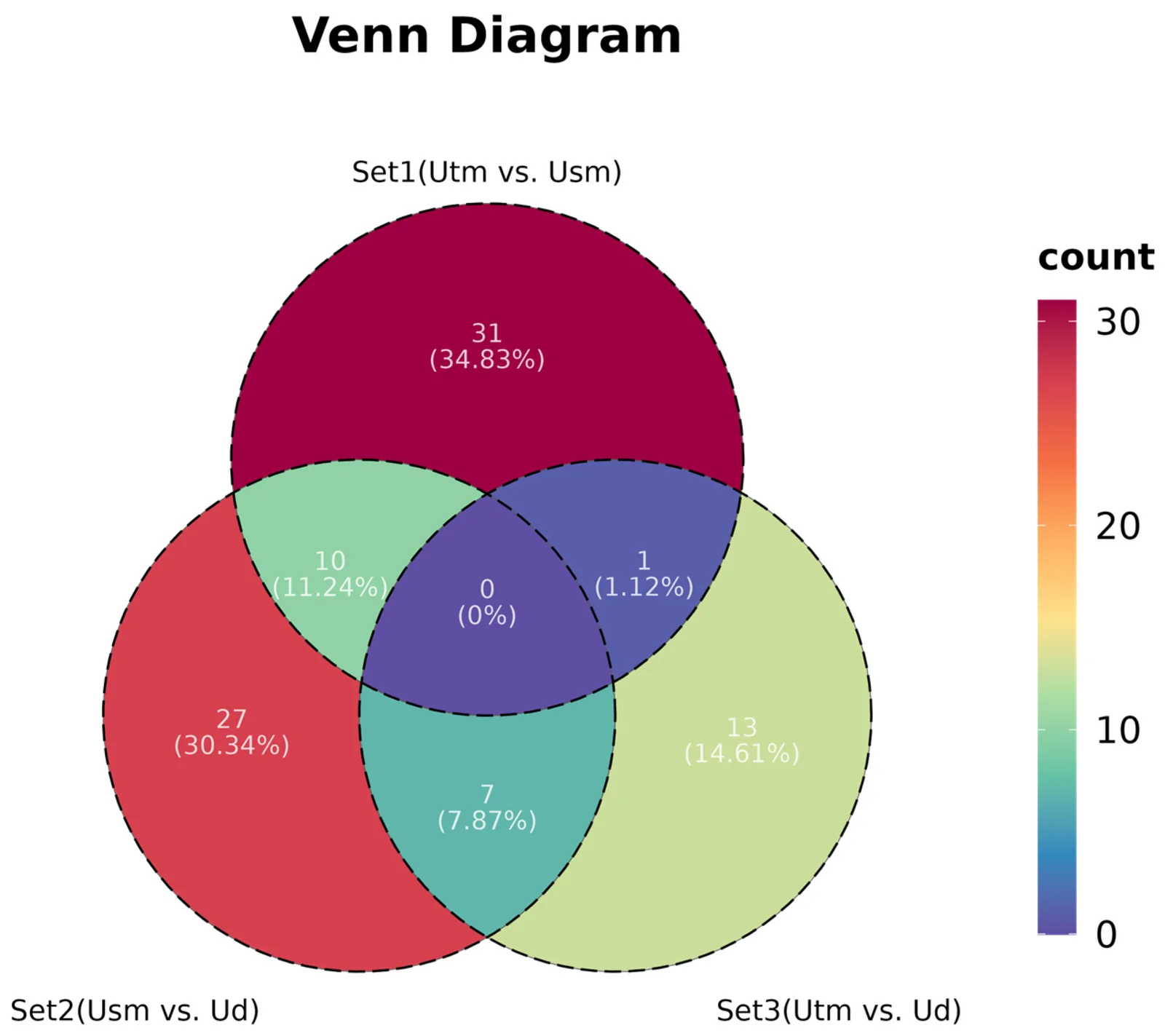
Venn diagram of DEMs among Utm vs. Usm vs. Ud.

To further dissect the relationships among the DEMs identified across the three comparison sets, we performed Venn diagram analysis (Figure 2). The results revealed that Set 1 and Set 2 shared 10 DEMs; Set 2 and Set 3 shared 7 DEMs; and Set 1 and Set 3 shared only 1 DEM. This intersection pattern carries clear biological stratification. The 10 DEMs in the Set 1 ∩ Set 2 intersection exhibited expression changes in both developmental transitions (3 → 6 months and 6 → 12 months) but failed to reach the significance threshold in the direct 3 → 12 months comparison. This pattern suggests that these miRNAs may be subject to stage-associated regulation. In contrast, the 7 DEMs in Set 2 ∩ Set 3 displayed a “late-onset, sustained into adulthood” pattern, characteristic of maturation-specific regulation, whereas the single DEM in Set 1 ∩ Set 3 followed an “early-onset, sustained into adulthood” pattern, indicative of early-established and persistent regulation. The absence of any DEM shared across all three sets (triple intersection = 0) indicates that no miRNA exhibited a continuous, monotonic linear trajectory throughout the entire developmental course. Collectively, these findings suggest that ovarian development is not governed by a single monotonous molecular trajectory, but rather by multiple temporally overlapping yet functionally distinct regulatory modules acting in concert.

### 3.3. Mfuzz Time-Series Clustering Identifies the “Transient Pulse” Pattern

**Figure 3:**
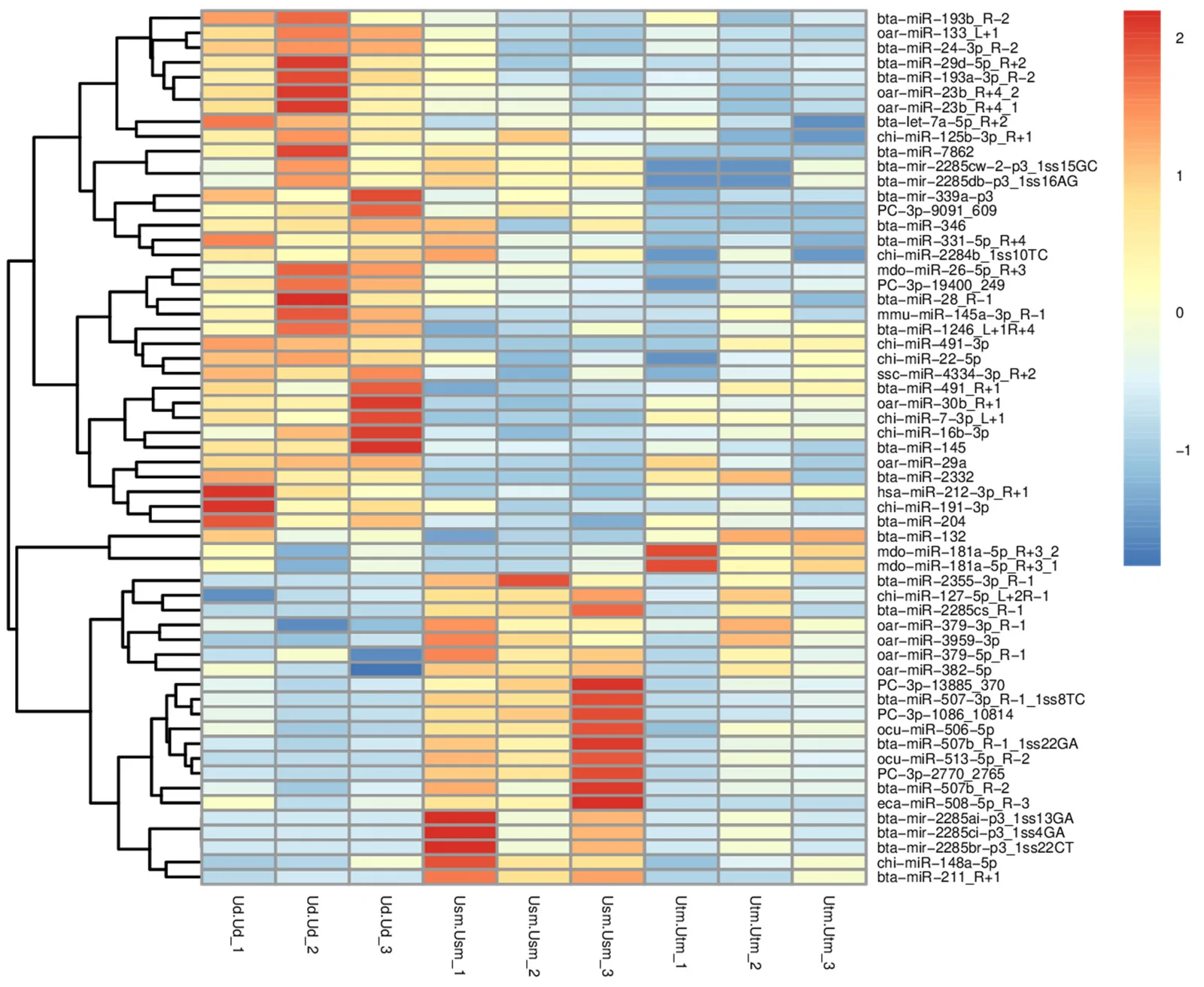
Heatmap of DEMs among Utm, Usm and Ud.

To systematically capture the dynamic changes in miRNA expression during ovarian development in Ujumqin sheep, we performed a global differential test using one-way ANOVA on the miRNA expression profiles across the three time points (Utm, Usm, and Ud). This analysis identified 59 miRNAs exhibiting significant overall expression differences among the three stages (Figure 3). The ANOVA results globally confirmed that the miRNA expression profiles differed significantly in a time-dependent manner across ovarian developmental stages, corroborating the pairwise comparison findings.

**Figure 4:**
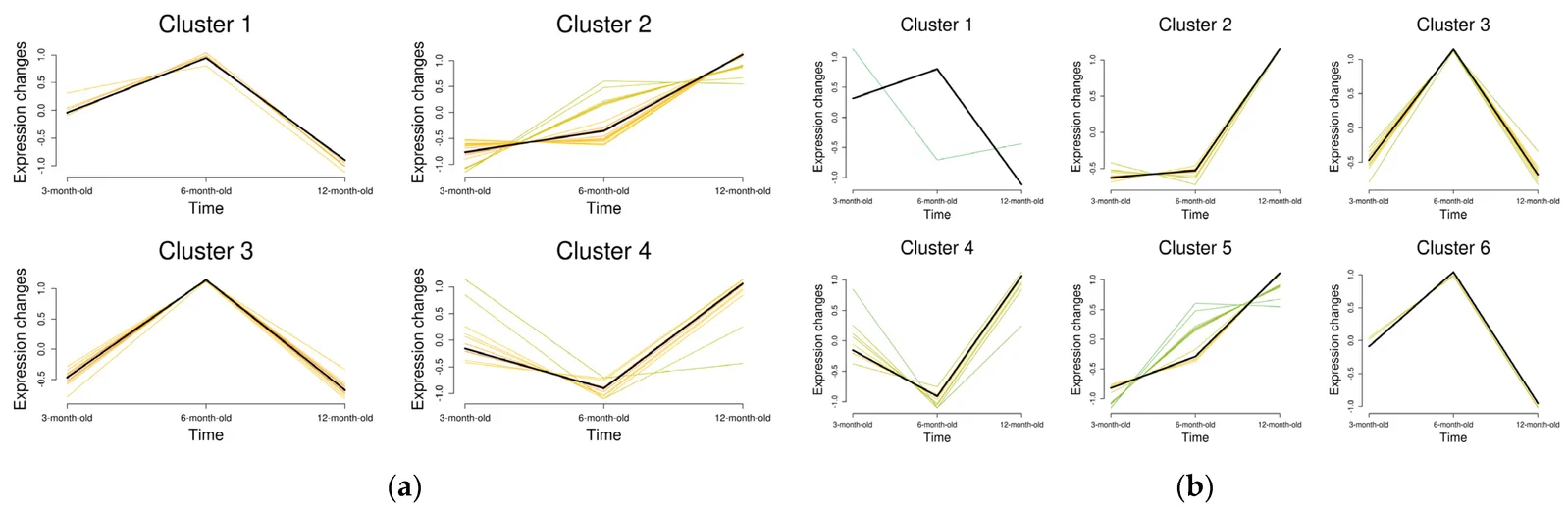
Mfuzz clustering of DEMs across Utm vs. Usm vs. Ud. (**a**): c = 4; (**b**): c = 6.

To visualize the temporal expression patterns of these 59 core miRNAs across the three time points, we performed trend analysis using Mfuzz fuzzy C-means clustering. To evaluate the robustness of the clustering results and to identify potential fine-grained expression subtypes, we conducted parallel analyses with cluster numbers set to c = 4 and c = 6. The c = 4 clustering results (Figure 4a) partitioned the 59 miRNAs into four trend clusters, among which Cluster 3 exhibited a “rise-then-fall” trajectory—representing miRNAs that peaked in expression at the 6-month puberty initiation window and returned to baseline by 12 months, i.e., a “transiently activated” type. When we mapped the 10 candidate miRNAs from the Set 1 ∩ Set 2 Venn intersection onto the c = 4 clustering results, 7 of them were found to belong to Cluster 3, preliminarily suggesting an association between these transient-pulse candidate miRNAs and the rise–fall trend. The c = 6 clustering results further refined the above classification (Figure 4b), dividing the 59 miRNAs into six trend clusters. Notably, the original Cluster 3 (rise–fall type) from the c = 4 analysis was clearly split into two sub-clusters with distinct fluctuation amplitudes: Cluster 3 displayed a “rise-then-fall” pattern with moderate fluctuation, whereas Cluster 6 also exhibited a “rise-then-fall” trajectory but with a more dramatic amplitude.

The most critical finding emerged when we mapped the 10 transient-pulse candidate miRNAs (Set 1 ∩ Set 2) onto the c = 6 clustering results: all 10 miRNAs (100%) were assigned exclusively to Cluster 3. This assignment was consistent with two preceding observations: (1) pairwise differential analyses showed that these 10 miRNAs reached significance in both transitional windows (3 → 6 months and 6 → 12 months); and (2) the Venn intersection defined their transient property—differentially expressed in the transitional stages but not in the full-course comparison. In addition, the c = 6 clustering placed all 10 miRNAs into the rise-then-fall trend, specifically within Cluster 3, which showed moderate fluctuation amplitude. The overlap rate improved from 7/10 in the c = 4 analysis to 10/10 in the c = 6 analysis, indicating that increasing the cluster number enabled a more precise dissection of heterogeneity within the rise-then-fall trend and distinguished these miRNAs from other transiently activated miRNAs with larger fluctuation amplitudes (Cluster 6). Collectively, these results describe a coherent expression pattern for the 10 miRNAs and provide a framework for their downstream analysis (Membership scores for each miRNA are provided in Supplementary Table S6).

### 3.4. Functional Enrichment Analysis of Target Genes of Transient-Pulse Core miRNAs

**Figure 5:**
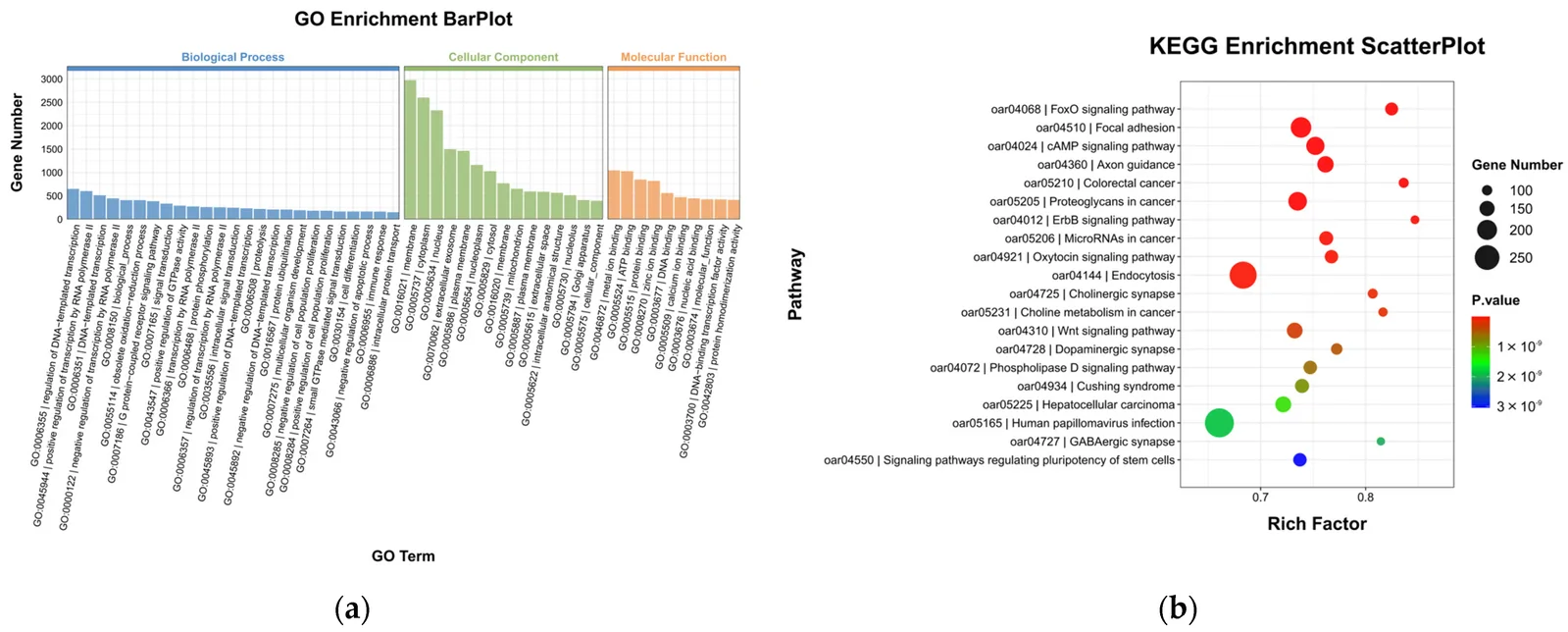
Functional enrichment analysis. (**a**): GO enrichment analysis; (**b**): KEGG pathway enrichment analysis.

To elucidate the biological functions of the 10 “transient-pulse core miRNAs” (miR-211, miR-29b-1-p3, miR-513-5p, PC-3p-1086, miR-507-3p, PC-3p-2770, miR-148a-5p, miR-379, miR-320d, and oar-miR-379-5p), we performed target gene prediction followed by GO and KEGG pathway enrichment analyses (Figure 5). The results revealed that the target genes were significantly enriched in pathways related to follicular development and ovulation regulation, including the Wnt and ErbB signaling pathways; hormone synthesis and secretion, such as the FoxO and cAMP signaling pathways; and the oxytocin signaling pathway. These enrichment results suggest that these 10 miRNAs are associated with molecular programs related to follicular development and hormone signaling at the 6-month puberty initiation window. At the adult stage (12 months), no significant differences were detected for these miRNAs between 3 and 12 months, and the corresponding signaling pathways may be less active, consistent with a relatively quiescent ovarian state.

### 3.5. Construction of the ceRNA Regulatory Network

**Figure 6:**
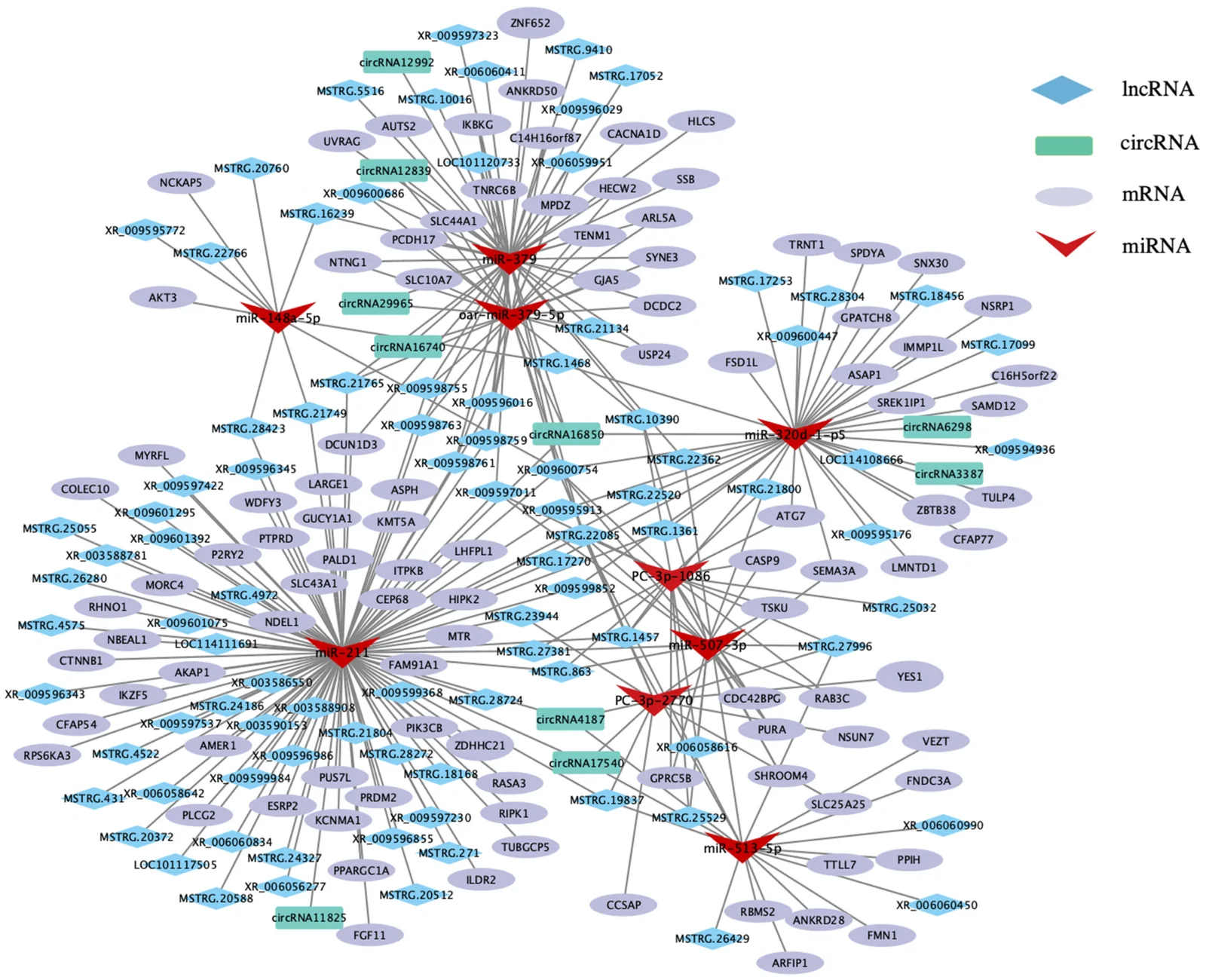
ceRNA regulatory network.

The above screening and comparative analyses have pinpointed 10 transient-pulse core miRNAs as the most dynamically responsive molecular players during the puberty initiation and sexual maturation transition windows in Ujumqin sheep, with their target pathways predominantly converging on the activation of follicular development and acute hormonal responses. To further dissect the upstream regulatory mechanisms governing these 10 miRNAs during the developmental window, we constructed a predicted ceRNA regulatory network centered on these miRNAs based on the ceRNA hypothesis. First, target mRNAs of the 10 core miRNAs were predicted using TargetScan and miRanda databases, and the intersection of the two prediction sets was further overlapped with DEMs in ovarian tissue, yielding 127 high-confidence predicted mRNA targets. Subsequently, based on this target mRNA set, we predicted circRNAs and lncRNAs that could potentially interact with the core miRNAs. The final predicted ceRNA network comprised 154 lncRNAs, 20 circRNAs, 9 miRNAs, and 127 mRNAs (Figure 6).

Network topology analysis showed that miR-211, miR-379, and miR-320d had the highest degrees of connectivity within the predicted network. The predicted target mRNAs of these miRNAs included CTNNB1, RPS6KA3, AUTS2, and ATG7, among others, and were enriched in pathways such as the Wnt signaling pathway, autophagy, and the TGF-β signaling pathway—findings that corroborate the KEGG enrichment results obtained for the transient-pulse core miRNAs. Further analysis of the ceRNA network pinpointed two core regulatory axes: lncRNA MSTRG.20588-miR-211-CTNNB1 and circRNA3387-miR-320d-ATG7. Given the established roles of CTNNB1 and ATG7 in follicular development and in the selection/elimination of follicular quality, respectively, these predicted axes suggest a hypothesis that Ujumqin sheep may use ceRNA interaction modules during the puberty initiation window to modulate granulosa cell responsiveness to gonadotropins, which may contribute to the molecular underpinnings of their seasonal, single-litter breeding phenotype.

### 3.6. PPI Network Topological Analysis and Core Gene Identification

**Figure 7:**
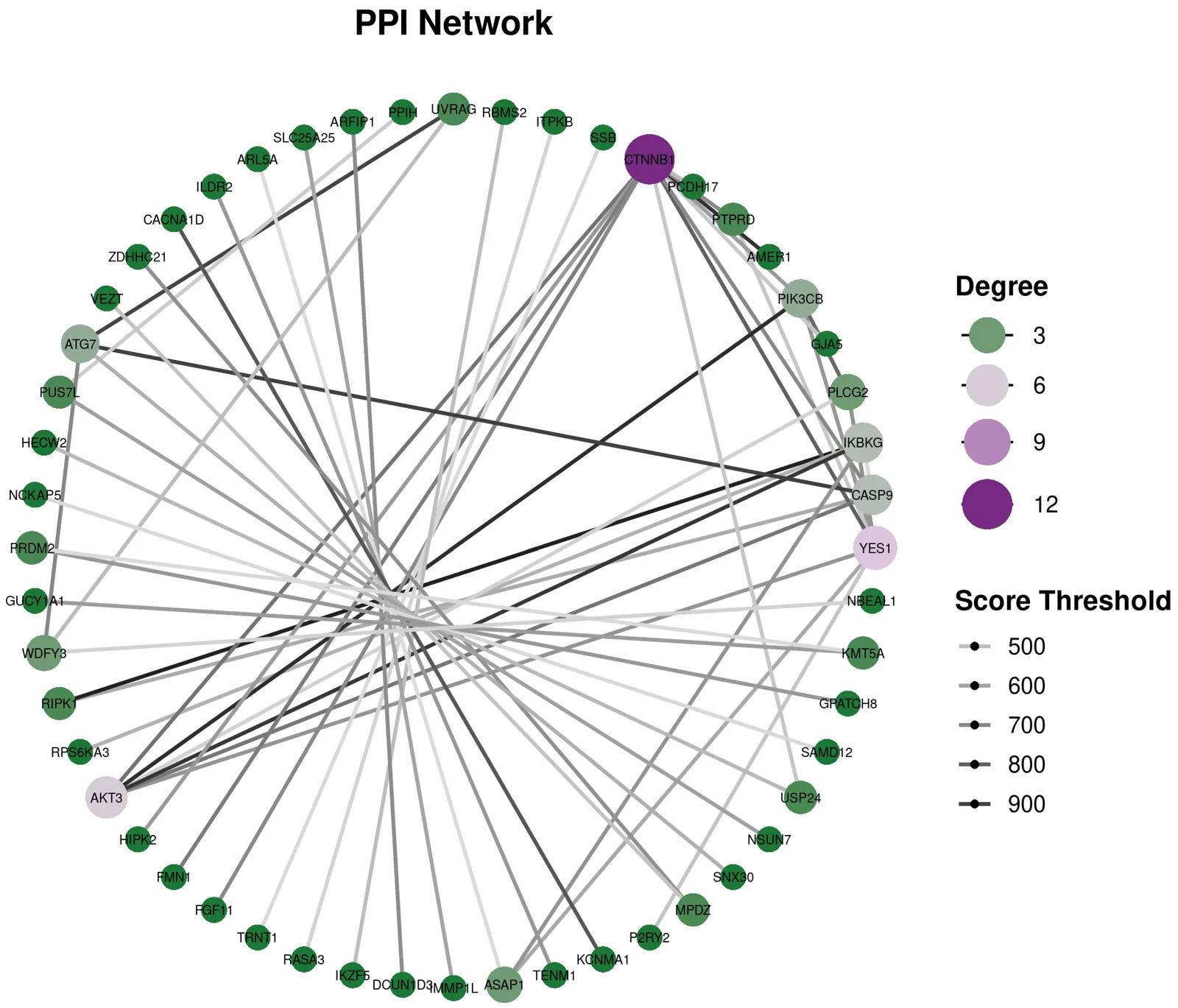
PPI Network Analysis.

To evaluate the functional associations among the target gene products at the protein level, we mapped the target genes of the transient-pulse core miRNAs onto the STRING database. Following confidence filtering (>0.4), a PPI network comprising 102 nodes and 51 interaction pairs was constructed. Within this network, CTNNB1, YES1, and AKT3 exhibited the highest node degrees (Figure 7).

### 3.7. Comparative Analysis of miRNA Dynamic Changes During Ovarian Development Between Ujumqin and Small-Tailed Han Sheep

**Figure 8:**
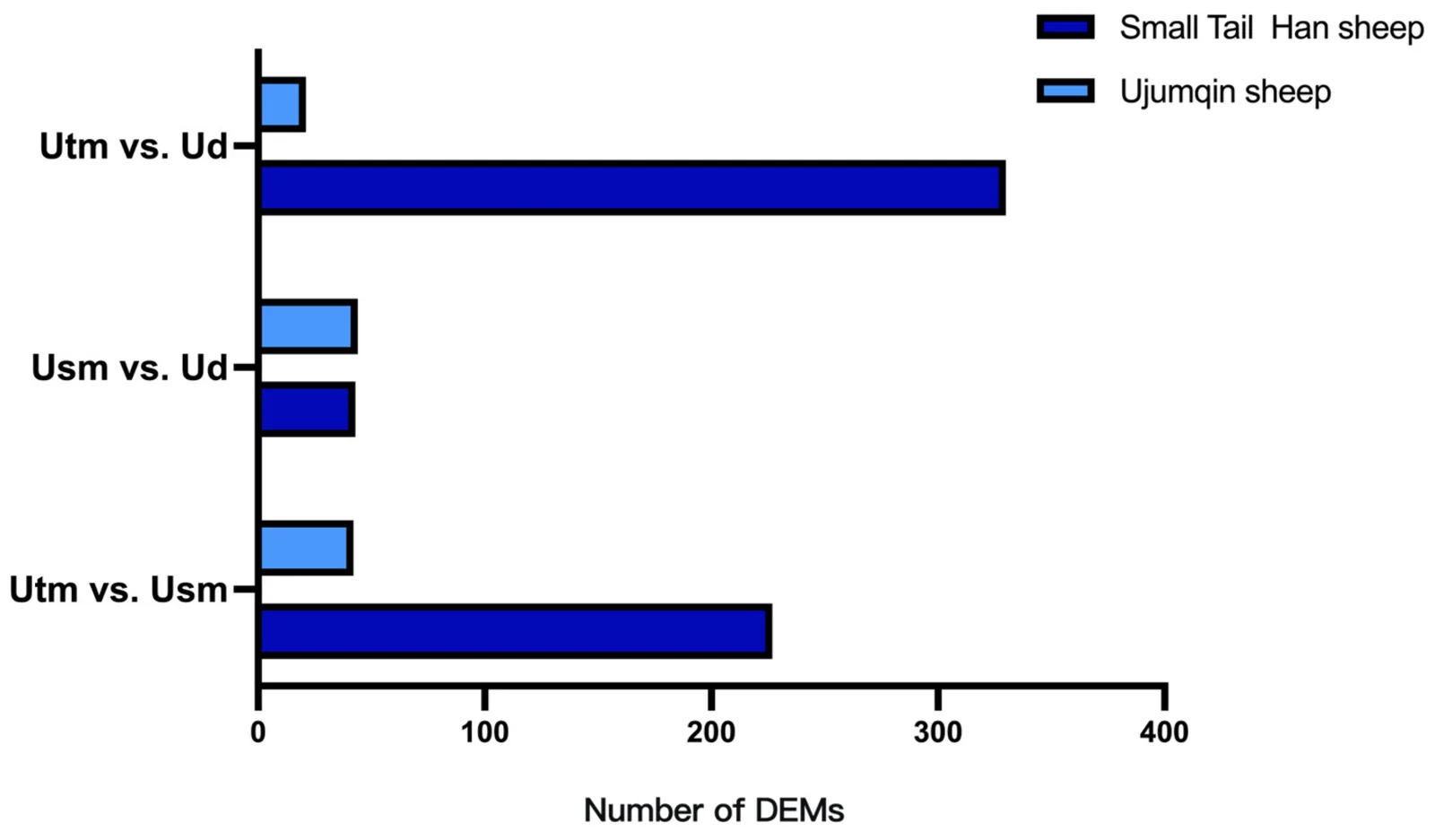
Numbers of DEMs between Small Tail Han sheep and Ujumqin sheep.

To further dissect the seasonal, monoestrous characteristics of Ujumqin sheep, we performed a cross-breed comparative analysis of the number of DEMs identified at the three developmental stages between Ujumqin sheep (a single-litter breed) and Small-Tailed Han sheep (a prolific breed), using data obtained previously in our laboratory for the corresponding temporal windows (Figure 8). It should be noted that the initial sampling time point for Small-Tailed Han sheep was 1 month of age, whereas that for Ujumqin sheep was 3 months of age; both time points represent the juvenile baseline state prior to the onset of sexual maturation. The remaining two time points (6 months and 12 months) were identical between the two breeds, ensuring the reliability of the mid-to-late stage comparisons.

The cross-breed comparison revealed that the prolific breed Small-Tailed Han sheep exhibited 227 DEMs between 1 and 6 months of age at the puberty initiation window, whereas Ujumqin sheep displayed only 42 DEMs between 3 and 6 months. Small-Tailed Han sheep therefore had approximately 5.4 times as many DEMs as Ujumqin sheep, suggesting that the prolific breed undergoes a more pronounced miRNA expression landscape remodeling upon entering puberty. During the post-maturation maintenance phase (6 to 12 months), the number of DEMs in the two breeds converged considerably: 43 in Small-Tailed Han sheep versus 44 in Ujumqin sheep. This near equivalence indicates that after reaching 6 months of age, the magnitude of miRNA fluctuations in ovarian tissue tends to converge regardless of litter size, potentially reflecting a species-conserved homeostatic regulatory mechanism operative at maturity. When considering the cumulative changes from the juvenile baseline to adulthood (baseline to 12 months), Small-Tailed Han sheep exhibited 330 DEMs between 1 and 12 months, whereas Ujumqin sheep displayed only 21 DEMs between 3 and 12 months—a more than 15-fold difference. Further examination of the relationship between cumulative and stage-specific changes revealed that the number of DEMs in Small-Tailed Han sheep (1 vs. 12 months, 330) approximately equaled the sum of those detected in 1 vs. 6 months (227) and 6 vs. 12 months (43), with only a modest additional overlap, indicating that the early changes were largely sustained into adulthood. In contrast, the number of DEMs in Ujumqin sheep (3 vs. 12 months, 21) was far smaller than the sum of those in 3 vs. 6 months (42) and 6 vs. 12 months (44), demonstrating that the majority of differential changes observed at 6 months were subsequently reversed in adulthood, ultimately returning to a molecular state resembling that of the juvenile stage. To further assess transcriptome reproducibility, we performed Pearson correlation and PCA analyses for each breed. As shown in Supplementary Figures S7 and S8, Ujumqin sheep showed a higher median replicate correlation and smaller within-group PCA distances, whereas Small-tailed Han sheep showed lower replicate correlations and larger within-group distances.

Collectively, these two breeds exhibited markedly distinct modes of miRNA dynamic regulation during ovarian development. In Ujumqin sheep, the ovary maintained a relatively stable state of transcriptomic rigidity throughout development. This rigidity may limit the ovary’s responsiveness to internal and external signals during the non-breeding season, a state that is consistent with the confinement of reproductive activity to the seasonal window. By contrast, Small-Tailed Han sheep (continuously breeding) establish a “transcriptomic plasticity” state during development, enabling year-round responsiveness to reproductive signals and supporting multiple ovulatory cycles per year. This divergence at the transcriptomic regulatory level provides a conceptual entry point for further exploration of the molecular basis underlying breed differences in fecundity.

### 3.8. RT-qPCR Validation

**Figure 9:**
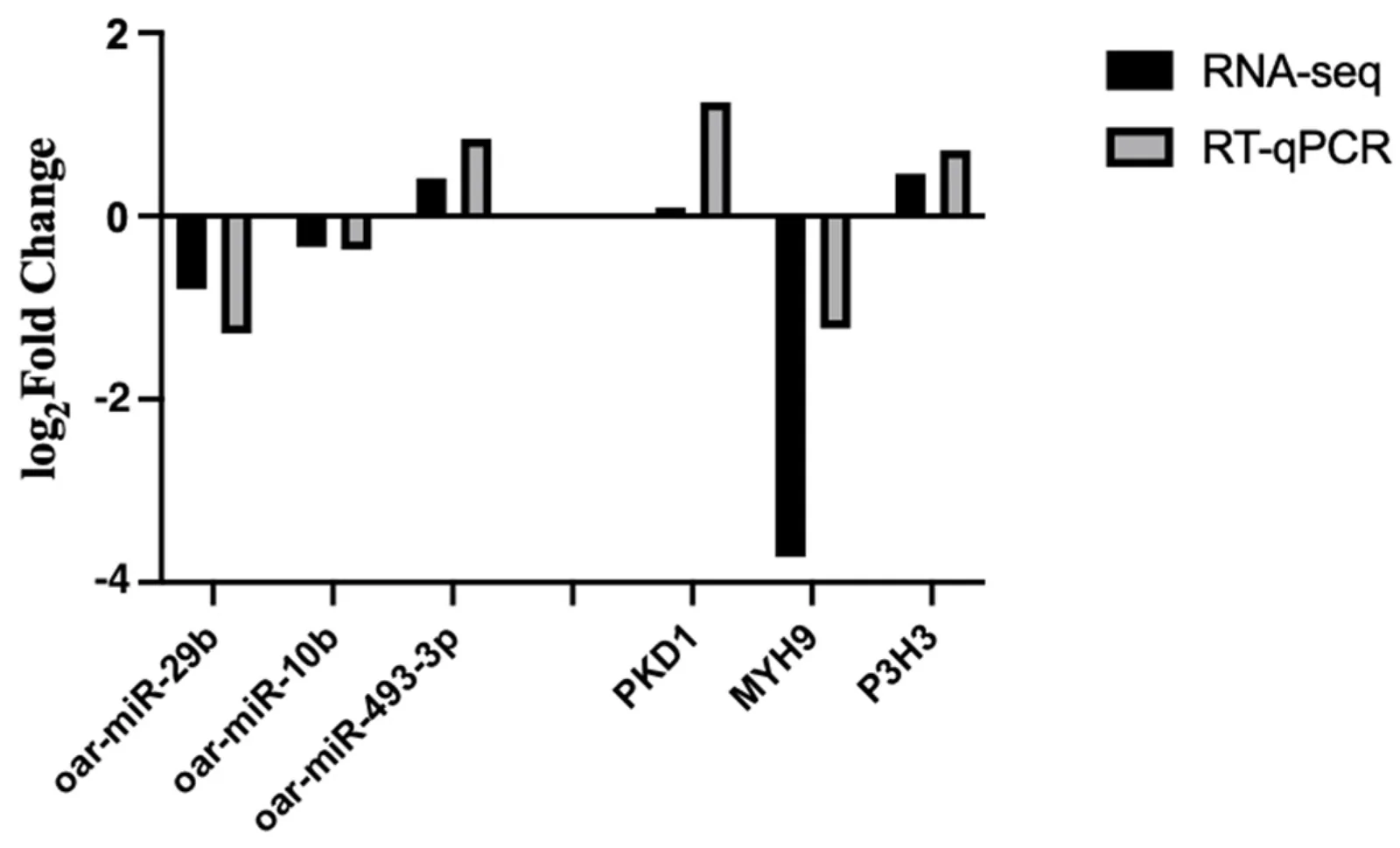
RT-qPCR validation of the RNA-Seq data.

To validate the reliability of the whole-transcriptome sequencing data, three randomly selected miRNAs (oar-miR-29b, oar-miR-10b and oar-miR-493-3p) and three randomly selected mRNAs (PKD1, MYH9 and P3H3) were subjected to RT-qPCR analysis. The direction of expression change for these molecules, as determined by RT-qPCR, was consistent with that from RNA-seq (Figure 9), confirming the accuracy and reproducibility of the sequencing results.

## 4. Discussion

### 4.1. The “Transient Pulse” Pattern of miRNAs During Ovarian Development in Ujumqin Sheep and Its Biological Implications

In this study, we performed whole-transcriptome sequencing of ovarian tissues from Ujumqin sheep at distinct developmental stages and systematically mapped the dynamic expression landscapes of miRNAs, lncRNAs, circRNAs, and mRNAs. Our findings revealed that the DEMs during ovarian development in Ujumqin sheep did not follow a monotonic increasing or decreasing trajectory from the pre-pubertal stage through puberty initiation to sexual maturity. Instead, it exhibited a highly nonlinear “transient pulse” pattern. Pairwise differential comparisons yielded 42 DEMs in Utm vs. Usm and 44 DEMs in Usm vs. Ud, yet only 21 DEMs in Utm vs. Ud—substantially fewer than either transitional comparison and markedly lower than conventional expectations based on phenotypic divergence. This “inverted-V” distribution indicates that a large cohort of miRNAs activated during the puberty initiation window (3 → 6 months) are systematically “withdrawn” upon entry into adulthood (6 → 12 months), resulting in a smaller molecular divergence between the pre-pubertal and adult stages than between the pre-pubertal and pubertal stages. This non-monotonic dynamic pattern could easily be overlooked in conventional pairwise comparisons but was clearly visualized through Mfuzz time-series clustering analysis. When the cluster number was set to c = 6, the 10 transient-pulse candidate miRNAs from the Set 1 ∩ Set 2 intersection were all (100%) assigned to Cluster 3—a sub-cluster characterized by a moderate and stable fluctuation amplitude with a “rise-then-fall” trajectory. This result was validated through three independent lines of evidence: pairwise differential analyses confirmed that these 10 miRNAs reached significance in both transitional windows; Venn diagram intersection analysis defined their transient nature—differentially expressed only in the transitional stages but not in the full-course comparison; and unsupervised Mfuzz clustering independently grouped all 10 miRNAs into the “rise-then-fall” trend cluster with moderate fluctuation amplitude. This strategy of cross-validation using multiple complementary approaches substantially strengthens the robustness of our conclusions.

The biological significance of this “transient pulse” pattern warrants careful consideration. Puberty initiation represents a critical developmental turning point in the reproductive lifespan of female mammals, involving comprehensive activation of the hypothalamic–pituitary–ovarian (HPO) axis, recruitment of follicles from the resting pool, and completion of the first ovulation [32]. During such a dramatic physiological transition, miRNAs act as “molecular switches” that are rapidly upregulated to activate molecular programs related to follicular development and hormonal signaling, and swiftly return to baseline levels once the transition is accomplished—a strategy consistent with the concept of transcriptome “resetting” following key developmental transitions in biological systems [33]. Notably, our target gene functional enrichment analysis demonstrated that the targets of these 10 transient-pulse miRNAs were significantly enriched in the Wnt, ErbB, FoxO, cAMP, and oxytocin signaling pathways—all of which are universally implicated in folliculogenesis, hormone synthesis and secretion, and ovulation regulation. Among these, the Wnt signaling pathway plays a central role in granulosa cell proliferation and follicle dominance selection [34,35]; the ErbB pathway mediates LH signal transduction during ovulation and influences follicular development [36,37]; the FoxO signaling pathway exerts critical regulatory functions in granulosa cell proliferation, steroid hormone synthesis, and follicle fate determination [38,39,40]; and the cAMP signaling pathway serves as the core mediator of gonadotropin signal transduction [41,42,43]. Thus, the functional portrait of this transient-pulse miRNA cohort is highly coherent: they transiently activate the “molecular license” for follicular development at the 6-month puberty initiation window and are subsequently switched off upon completion of the task.

This finding suggests that in seasonally breeding, monoestrous breeds such as Ujumqin sheep, puberty initiation does not constitute an irreversible “developmental lock-in,” but rather resembles a reversible “molecular trial run”—the ovary does not permanently anchor its transcriptomic state to the adult configuration after completing puberty initiation, but instead reverts to a quiescent state approximating the pre-pubertal level. The evolutionary logic underlying this strategy may be rooted in the seasonal breeding pattern: during the non-breeding season, the ovary must remain in a “standby” rather than a “continuously activated” state, thereby conserving energy expenditure and avoiding the initiation of ovulation at an inopportune time of year.

### 4.2. Divergent Ovarian Transcriptomic Regulatory Strategies Between Single-Litter and Prolific Breeds: “Rigidity” Versus “Plasticity”

To further assess the breed-specificity of the molecular features observed in Ujumqin sheep, we performed a cross-breed comparison of miRNA dynamic patterns with data from Small-Tailed Han sheep (a prolific, continuously breeding breed) obtained previously in our laboratory at corresponding developmental stages. The comparison revealed that at the puberty initiation window (baseline → 6 months), Small-Tailed Han sheep exhibited 227 DEMs, whereas Ujumqin sheep displayed only 42 DEMs—the former being approximately 5.4-fold greater than the latter. At the cumulative change level (baseline → 12 months), Small-Tailed Han sheep yielded 330 DEMs, which roughly equaled the sum of stage-specific changes, indicating that the early alterations were largely sustained into adulthood. In contrast, Ujumqin sheep yielded only 21 DEMs, far fewer than the sum of stage-specific changes (42 + 44), implying that the majority of differential changes observed at 6 months were subsequently reversed and essentially “reset” by adulthood. This contrast uncovers a critical feature of transcriptomic regulation in Ujumqin sheep ovaries: after completing puberty initiation, the ovary tends to reset the transcriptome back to the baseline state. We conceptualize this as “transcriptomic rigidity”—a state in which the ovarian transcriptome exhibits a narrow range of responsiveness to external and internal signals, rapidly reverting to a quiescent level once the requisite developmental transition has been accomplished. In contrast, Small-Tailed Han sheep establish a state of “transcriptomic plasticity,” characterized by the ability to sustain molecular changes acquired during development, thereby providing a transcriptome-level elastic reserve that supports multiple estrous cycles and ovulations throughout the year.

Notably, this “rigidity” strategy in Ujumqin sheep aligns closely with their seasonal, monoestrous breeding characteristics. Under the natural photoperiod, Ujumqin sheep ovulate only during the autumnal breeding season when daylight shortens. Outside this window, in the absence of exogenous gonadotropin support, ovulation does not normally occur, and the ovary displays a relatively low-activity transcriptomic state [44]. The ovarian transcriptomic dynamics observed in this study may reflect this seasonal shift in ovarian activity under natural conditions. From the perspective of transcriptomic regulation, “rigidity” implies that the transcriptomic state is less susceptible to perturbation during the non-breeding season, thereby ensuring that the ovary’s responsiveness to external signals is confined within the specific seasonal window. In this sense, “transcriptomic rigidity” may represent the molecular hallmark of seasonally breeding breeds—it is not a “deficit” but rather an adaptive strategy. This conceptual framework also offers a novel perspective for understanding breed differences in fecundity. Previous comparisons between single-litter and prolific breeds have largely focused on differential expression of specific genes or pathways. The present study suggests, however, that a more fundamental divergence may reside at the level of global transcriptomic regulatory strategy—whether to adopt “transient pulses” or “sustained remodeling,” and whether to maintain “rigidity” or establish “plasticity.”

### 4.3. Potential Functions of Core miRNA–ceRNA Regulatory Axes

Among the ten transient-pulse core miRNAs, miR-211, miR-379, and miR-320d ranked top three in connectivity within the ceRNA network, serving as central hubs of this network. The known functions of these three miRNAs are highly consistent with the findings of this study.

miR-211 was identified as a key DEM between breeds in our previous study, with significantly higher expression in the ovaries of Small-Tailed Han sheep compared to Ujumqin sheep [45]. In ovarian function research, miR-211-5p has been shown to participate in ovarian repair and regulation through the MALAT1/miR-211-5p/FOXO3 axis [46]. In the present study, miR-211 was found to target CTNNB1 (β-catenin)—the core effector molecule of the Wnt signaling pathway [47]—and to form a ceRNA regulatory axis via lncRNA MSTRG.20588. Given the established roles of CTNNB1 in granulosa cell proliferation and follicle dominance selection [48,49], the identification of this axis suggests that Ujumqin sheep may employ specific ceRNA modules during the puberty initiation window to precisely modulate the responsiveness threshold of granulosa cells to gonadotropins. miR-379-5p has been reported to be closely associated with ovarian function—participating in cellular damage responses via PARP1 and XRCC6 in premature ovarian failure, and regulating granulosa cell fate in androgen-induced polycystic ovary syndrome [50]. In androgen-induced polycystic ovary syndrome, miR-379-5p has been shown to regulate granulosa cell fate through exosomal release, and its aberrant expression is linked to follicular growth arrest [51]. In the present study, miR-379 occupies a central hub position within the ceRNA network, suggesting that it may play a role in the molecular regulation of puberty initiation that is far more critical than previously appreciated. miR-320d targeted ATG7—a key gene in the autophagy pathway [52]—and formed a regulatory axis via circRNA3387. The role of autophagy in follicular development and oocyte maturation has garnered extensive attention in recent years [53], and ATG7-mediated autophagic processes are involved in the selection and elimination of follicular reserves [54,55]. The identification of this axis directly links the transient-pulse miRNAs to the cellular processes of follicular quality control.

The identification of the above ceRNA axes carries two levels of significance. First, they provide a mechanistic explanation for the upstream regulation of the transient-pulse miRNAs—lncRNAs and circRNAs modulate the availability of miRNAs through “sponge” adsorption, thereby fine-tuning miRNA activity at the post-transcriptional level. Second, these axes integrate miRNAs, mRNAs, and non-coding RNAs into functional modules rather than as isolated molecular events—an observation consistent with the contemporary view that ovarian development is driven by the coordinated action of multilayered regulatory networks [56,57]. It is worth emphasizing that both ceRNA axes—lncRNA MSTRG.20588–miR-211–CTNNB1 and circRNA3387–miR-320d–ATG7—are centered on regulatory events occurring at the 6-month puberty initiation window in Ujumqin sheep. The target genes involved (CTNNB1 and ATG7) are both well-established key factors in follicular development and follicular quality control, suggesting that the molecular regulation of puberty initiation in Ujumqin sheep does not entail “coarse” genome-wide activation, but rather achieves precise regulation of key node genes through a limited number of core ceRNA modules. This study has several limitations. First, the ceRNA network and miRNA–target relationships are based on transcriptome-level correlation analysis and database predictions and have not been functionally validated. Future work should use dual-luciferase reporter assays, granulosa cell overexpression/knockdown, and in vitro culture systems to verify the regulatory relationships of the core ceRNA axes and their effects on granulosa cell proliferation, apoptosis, and hormone secretion. Second, the sample size was small (n = 3 per time point), limiting statistical power. Sampling was age-based and conducted under the natural photoperiod, so developmental age, photoperiodic history, and seasonal reproductive state were confounded. This study should therefore be viewed as a developmental time-course in a seasonal breed, not as a test of photoperiod-driven transitions. Future studies with controlled photoperiod and repeated sampling at the same age are needed to isolate photoperiod effects. In addition, LH pulse frequency and amplitude were not directly measured in this study; the ovarian miRNA dynamics may reflect downstream responses to changes in LH secretion from puberty initiation to sexual maturity. Future work combining serial LH sampling with ovarian transcriptomics will help clarify how central neuroendocrine signals are relayed to the ovary in Ujumqin sheep. These limitations also provide directions for future research.

## 5. Conclusions

In this study, we performed whole-transcriptome sequencing to reveal a “transient pulse” pattern of miRNA expression during ovarian development in Ujumqin sheep, a seasonally breeding, monoestrous breed. This pulse was activated at the puberty initiation window and subsided by sexual maturity. Through triple cross-validation integrating Mfuzz clustering and Venn diagram intersection analyses, we pinpointed 10 core miRNAs whose target genes were significantly enriched in pathways critical for follicular development, including Wnt, cAMP, and FoxO signaling. Construction of a ceRNA network further anchored two core regulatory axes—lncRNA MSTRG.20588-miR-211-CTNNB1 and circRNA3387-miR-320d-ATG7—directly linking the pulse signals to follicle dominance selection and autophagy-mediated follicular quality control. Cross-breed comparison revealed that the prolific breed Small-Tailed Han sheep exhibited a miRNA response magnitude 5.4-fold greater than that of Ujumqin sheep at the puberty initiation window, with the changes largely sustained into adulthood, whereas in Ujumqin sheep, the molecular perturbations observed at 6 months were systematically reset by adulthood. Based on these findings, we propose a conceptual framework distinguishing “transcriptomic rigidity” versus “transcriptomic plasticity” between breeds: the ovary of Ujumqin sheep tends to reset its transcriptome to a quiescent state after completing puberty initiation—a strategy that may represent the molecular hallmark of seasonally breeding, monoestrous breeds. This suggests that the monoestrous trait is not simply a deficit in gene expression, but rather a coordinated transcriptomic adaptive strategy. Collectively, this study provides a new conceptual framework and actionable candidate targets for deciphering the molecular mechanisms underlying seasonal reproduction in sheep, taking an important step toward ultimately unlocking the molecular code of seasonal breeding.

## Data Availability

The raw RNA-seq data generated in this study have been deposited in the NCBI Sequence Read Archive (SRA) under the BioProject accession number PRJNA1526712.

## Supplementary Materials

The following supporting information can be downloaded at https://www.mdpi.com/article/10.3390/biology15201828/s1, Figure S1: Expression profile characteristics of lncRNAs and mRNAs. (a): Coverage depth profiling of transcripts; (b): lncRNA exon number distribution; Figure S2: Expression profile characteristics of miRNAs. (a): Length distribution of clean reads from identified miRNA fragments.; (b): Bias distribution of the first base of miRNA sequence; Figure S3: Expression profile characteristics of circRNAs. (a): circRNA type statistics; (b): circRNA sequence length; Figure S4: Volcano plot of differentially expressed circRNAs. (a): Utm vs. Usm; (b): Usm vs. Ud; (c): Utm vs. Ud; Figure S5: Volcano plot of differentially expressed lncRNAs. (a): Utm vs. Usm; (b): Usm vs. Ud; (c): Utm vs. Ud; Figure S6: Volcano plot of differentially expressed mRNAs. (a): Utm vs. Usm; (b): Usm vs. Ud; (c): Utm vs. Ud; Figure S7: Pearson correlation heatmap of miRNA expression profiles. (a): Ujumqin sheep; (b): Small-tailed Han sheep; Figure S8: PCA of miRNA expression profiles. (a): Ujumqin sheep; (b): Small-tailed Han sheep; Table S1: RT-qPCR primers sequence information; Table S2: Detection results of total RNA quality; Table S3: mRNA and lncRNA sequencing data production quality; Table S4: sRNA sequencing data production quality; Table S5: fold_change and norm for the 10 core miRNAs; Table S6: Mfuzz clustering results of the 10 core miRNAs.

## Abbreviations

The following abbreviations are used in this manuscript:

DEMs	differentially expressed miRNAs
DELs	differentially expressed lncRNAs
DECs	differentially expressed circRNAs
ncRNAs	non-coding RNAs
