# Supplementary material for: Transient Pulses and Transcriptomic Rigidity: Dynamic miRNA Expression Patterns in Ovine Ovarian Development Reveal Molecular Strategies Underlying Seasonal Reproduction
Source: Biology (Basel). 2026 Oct 13;15(20):1828. doi: 10.3390/biology15201828 (PMC13701545; doi:10.3390/biology15201828)
Supplement: Supplementary file 1 [file biology-15-01828-s001.zip › biology-4589464-supplementary.pdf]

Table S1 RT-qPCR primers sequence information

Genes	Primer sequences(5'→3')	Fragment length/bp
β-actin	F: GATCTGGCACCACACCTTCTA	115
	R: GATCTGGGTCATCTTCTCACG	
PKD1	F:CGCTCATCGCTGTGCTCAAC	112
	R:ACCAATGTCTCCGTGATGTTCTTG	
MYH9	F: TCACCAAGGACTTCTCTGCTCTG	120
	R:TCTTCTCATCCTCCACCTGCTTC	
P3H3	F:GGTGGCTGGAGGTGGGAGAAG	125
	R:GCATGAGGAAGGCAGGTGGTTG	
U6	F:ATCCAGTGCAGGGTCCGAGG	65
	R:TGAAGCGTGCTCGCTTCGGC	
oar-miR-29b	F:CTCAACTGGTGTCGTGGAGTCGGCAATTCA GTTGAGACACTGAT	66
	R:ACACTCCAGCTGGGTAGCACCATTTGAAAT	
oar-miR-10b	F:CGCACCCTGTAGAACCGAATTTGTG	65
	R:CACAAATTCGGTTCTACAGGGTGCG	
oar-miR-493-3p	F:TGAAGGTCTACTGTGTGCCAGG	65
	R:CCTGGCACACAGTAGACCTTCA	

Table S2 Detection results of total RNA quality

Samples	concentration (μg/μL)	RIN	OD value(260/280)
Utm_1	0.4880	7.90	1.85
Utm_2	0.0303	7.00	1.82
Utm_3	0.0542	8.20	1.96
Usm_1	0.0350	7.00	1.81
Usm_2	0.0531	7.10	1.83
Usm_3	0.0462	7.40	1.87
Ud_1	0.4903	8.20	1.83
Ud_2	0.0501	7.50	1.89
Ud_3	0.0718	10.00	2.00

Table S3 mRNA and lncRNA sequencing data production quality

Sample	Raw Data	Valid Data	Mapping rate（%）	Q20/%	Q30/%	GC content%
Utm_1	80,012,692	77,430,674	70,474,898(91.02%)	99.85	97.94	47.00
Utm_2	85,672,174	83,316,694	77,529,678(93.05%)	99.84	97.94	47.50
Utm_3	76,370,866	74,060,470	69,179,061(93.41%)	99.84	97.77	46.00
Usm_1	87,546,078	84,987,084	79,180,099(93.17%)	99.84	97.94	45.00
Usm_2	92,533,792	89,836,724	85,036,480(94.66%)	99.83	98.15	45.00
Usm_3	82,541,404	80,135,794	74,815,028(93.36%)	99.84	97.93	45.50
Ud_1	73,320,580	70,898,902	65,359,324(92.19%)	99.85	97.83	47.00
Ud_2	92,249,184	87,955,300	82,065,791(93.30%)	99.77	97.86	50.50
Ud_3	71,516,612	68,959,698	63,802,517(92.52%)	99.81	97.60	48.00

Table S4 sRNA sequencing data production quality

Sample	Raw Data	Valid Reads	Mapping rate（%）	Q20/%	Q30/%	GC content%
Utm_1	9,924,884	9,172,255	8107357 (88.39%)	99.16	96.86	56.88
Utm_2	11,584,677	10,980,229	10895682 (99.23%)	99.13	96.89	50.79
Utm_3	10,229,692	9,711,195	9641275 (99.28%)	99.20	97.21	51.09
Usm_1	13,061,831	12,267,972	12147746 (99.02%)	99.22	97.10	50.93
Usm_2	10,321,524	9,810,771	9736210 (99.24%)	99.33	97.29	50.91
Usm_3	10,955,021	10,352,222	10260088 (99.11%)	99.33	97.41	50.98
Ud_1	11,079,361	10,447,964	10376918 (99.32%)	99.24	97.13	51.14
Ud_2	11,405,958	10.843,603	10768783 (99.31%)	99.26	97.10	50.87
Ud_3	11,156,801	10,668,896	10572876 (99.10%)	99.28	97.18	50.94

Table S5 fold_change and norm for the 10 core miRNAs.

	Norm (Utm)	Norm (Usm)	Norm (Ud)	fold_change (Utm vs. Usm)	fold_change (Usm vs. Ud)	fold_change (Utm vs. Ud)
miR-211	86.96	274.24	74.86	0.32	3.66	1.16
miR-29b-1-p3	0.00	6.78	0.00	-inf	inf	0.00
miR-513-5p	5.46	33.85	0.00	0.16	inf	inf
PC-3p-1086	257.29	1168.52	262.51	0.22	4.45	0.98
miR-507-3p	500.96	2144.12	447.83	0.23	4.79	1.12
PC-3p-2770	126.49	541.86	63.52	0.23	8.53	1.99
miR-148a-5p	360.60	427.92	356.63	0.84	1.20	1.01
miR-379	0.00	9.81	0.00	-inf	inf	0.00
miR-320d	0.00	3.52	0.00	-inf	inf	0.00
oar-miR-379-5p	6334.77	8768.22	5398.69	0.72	1.62	1.17

Table S6 Mfuzz clustering results of the 10 core miRNAs.

miRNA ID	C=4	Membership(c=4)	C=6	Membership(c=6)
miR-211	Cluster3	0.4984	Cluster3	0.3739
miR-29b-1-p3	Cluster1	0.3612	Cluster3	0.2908
miR-513-5p	Cluster3	0.4986	Cluster3	0.3751
PC-3p-1086	Cluster3	0.4325	Cluster3	0.3143
miR-507-3p	Cluster3	0.4620	Cluster3	0.3405
PC-3p-2770	Cluster3	0.5940	Cluster3	0.4703
miR-148a-5p	Cluster3	0.4907	Cluster3	0.3668
miR-379	Cluster1	0.3389	Cluster3	0.2418
miR-320d	Cluster1	0.6680	Cluster3	0.5603
oar-miR-379-5p	Cluster3	0.3652	Cluster3	0.2569

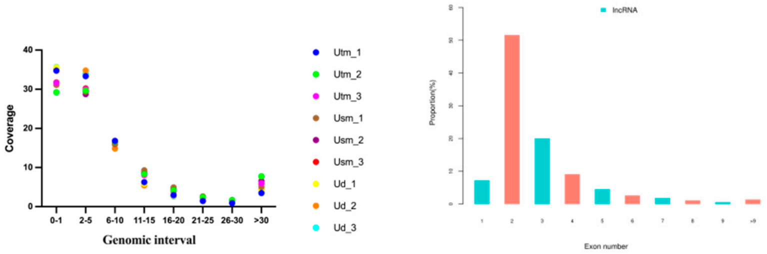

a b

Figure S1. Expression profile characteristics of lncRNAs and mRNAs. (a): Coverage depth profiling of transcripts; (b):lncRNA exon number distribution

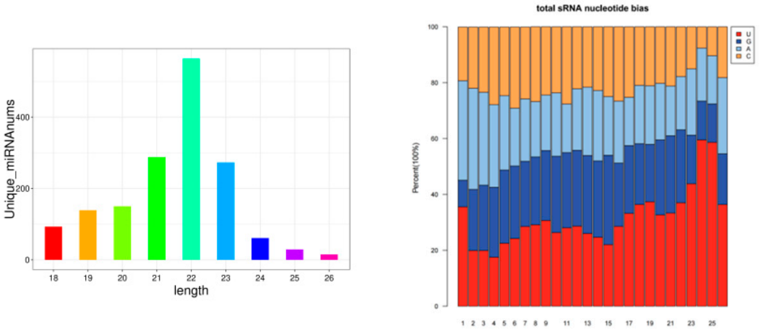

a b

Figure S2. Expression profile characteristics of miRNAs. (a): Length distribution of clean reads from identified miRNA fragments.; (b):Bias distribution of the first base of miRNA sequence

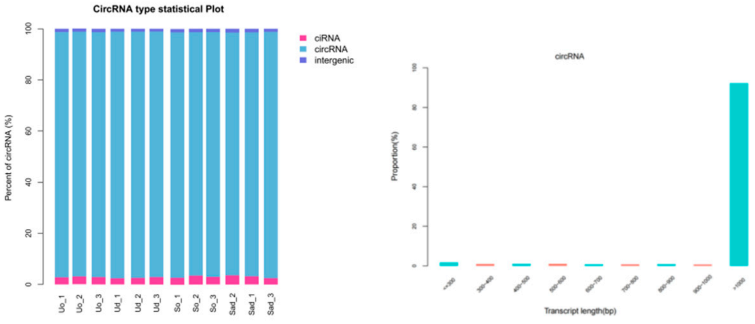

a **b**

Figure S3. Expression profile characteristics of circRNAs. (a): circRNA type statistics; (b): circRNA sequence length

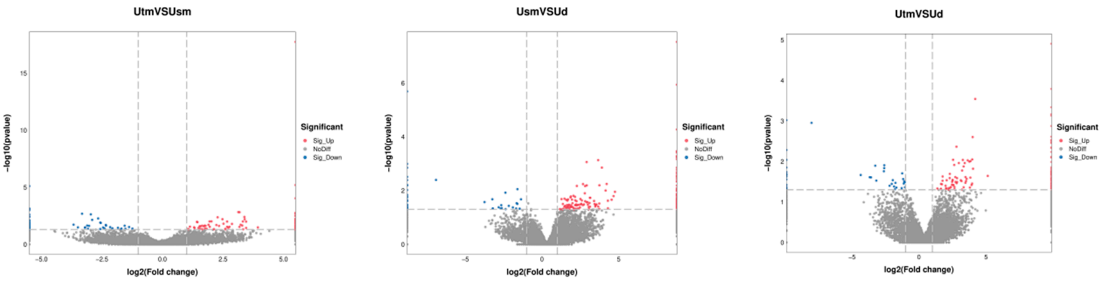

a b c

Figure S4. Volcano plot of differentially expressed circRNAs. (a): Utm vs. Usm; (b): Usm vs. Ud; (c): Utm vs. Ud

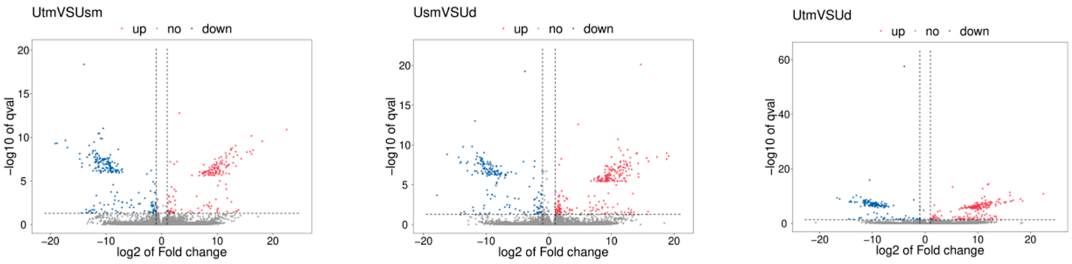

a b c

Figure S5. Volcano plot of differentially expressed lncRNAs. (a): Utm vs. Usm; (b): Usm vs. Ud; (c): Utm vs. Ud

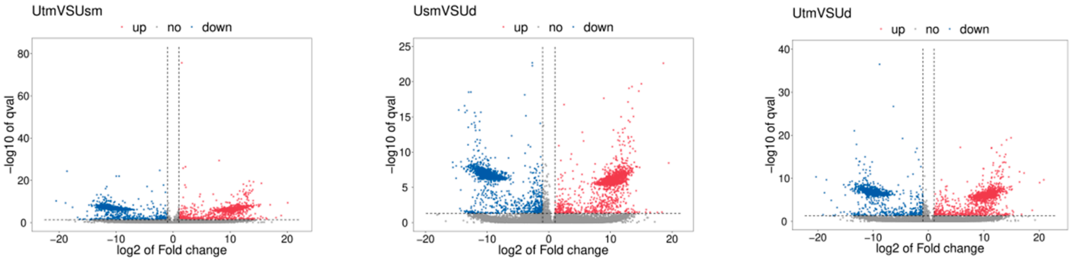

a b c

Figure S6. Volcano plot of differentially expressed mRNAs. (a): Utm vs. Usm; (b): Usm vs. Ud; (c): Utm vs. Ud

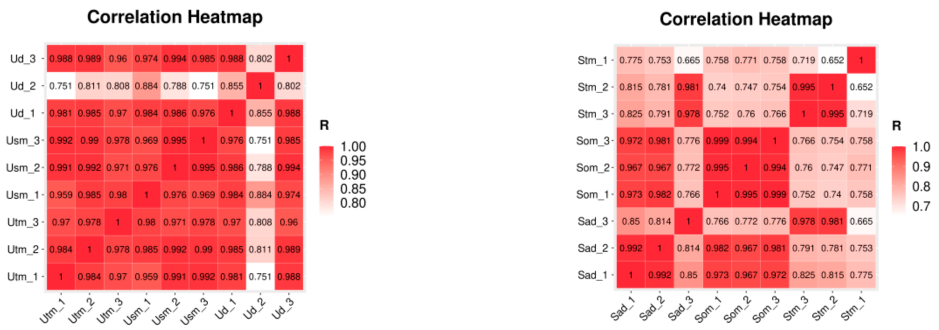

a b

Figure S7. Pearson correlation heatmap of miRNA expression profiles. (a): Ujumqin sheep; (b): Small-tailed

Han sheep

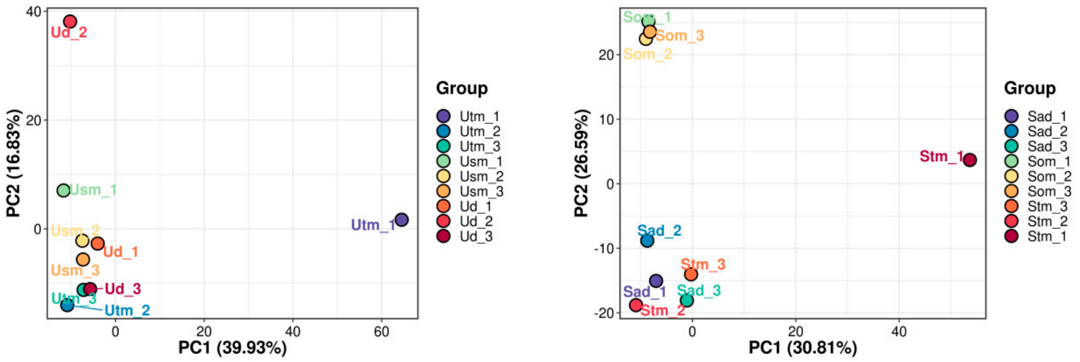

a b

Figure S8. PCA of miRNA expression profiles. (a): Ujumqin sheep; (b): Small-tailed Han sheep
